# Proteome and transcriptome reveal the involvement of heat shock proteins and antioxidant system in thermotolerance of *Clematis florida*

**DOI:** 10.1038/s41598-020-65699-2

**Published:** 2020-06-01

**Authors:** Changhua Jiang, Yuke Bi, Jianbin Mo, Ruyao Zhang, Mingnan Qu, Shucheng Feng, Jemaa Essemine

**Affiliations:** 1 Shanghai Botanical Garden, Shanghai, 200231 China; 2 CAS Center for Excellence in Molecular Plant Sciences, Institute of Plant Physiology and Ecology, Shanghai Institutes for Biological Sciences, Chinese academy of Sciences, Shanghai, 200032 China

**Keywords:** Heat, Plant physiology, C3 photosynthesis

## Abstract

*Clematis florida* Thun (*Cf*T) is an ornamental and medicinal plant. It is a cold resistant but heat sensitive species and deserves to be further investigated to improve its adaptability to heat stress. Exploring the molecular mechanism potential via an omic-analysis constitutes a promising approach towards improving heat tolerance of *Cf*T. Two *Cf*T lines, heat resistance (HR) and heat sensitive (HS), with differential thermotolerance capacities were used for the integrative analyses of proteomics and transcriptomes. Transcriptomes analysis showed that various pathways were significantly enriched including plant hormone signal transduction and carbon fixation pathways in prokaryotes. Proteomics study revealed the enrichment of some other pathways comprising antioxidant activity and carbohydrates metabolism. Based on combined transcriptomes and proteomics analyses and following heat stress treatment, a total of 1724 annotated genes were overlapped between both *Cf*T lines. Particularly, 84 differential expressed genes (DEGs) were overlapped in both *Cf*T lines. Fifteen out of these 84 genes were up-regulated solely for HR line (PS) but not for HS one (SG). This strongly suggests a potential prominent role for these genes in the thermotolerance process in PS line. We corroborate that two Hsps (Hsp18 and Hsp70) out of 20 detected proteins with higher expression levels in PS than in SG based on either global transcripts or proteins levels. According to the transcriptomes and proteomics analyses, 6 proteins and their corresponding genes were found to be significantly abundant in HR line (PS). Data are available via ProteomeXchange with identifier PXD018192. The expressions levels of these 6 genes were checked also for both *Cf*T lines to evaluate their potential contributions in the heat tolerance process. Thus, their expression levels were approximately 2~4 times higher in HR than in HS line. We provided as well a representative schematic model to highlight the key genes involved in ROS scavenging and photorespiratory pathway in *Cf*T. This model could be helpful also in understanding the mechanism of heat tolerance in *Cf*T.

## Introduction

*Clematis florida* Thun (*Cf*T) is known by its economic importance and can stimulate and activate the commercial sector since it represents an ornamental and medicinal plant. *Cf*T, called also “Queen of vines”, is a popular climbing plant worldwide. It has a high ornamental value and used in landscapes and floriculture as a garden or potted plant^1,2^. Most clematis varieties are bred in high latitudes or cold regions and are cold resistant but heat sensitive^2^. During summer season, temperatures ranging from 30~40 °C lead to constrained growth, twig wilting, leaf wilting, shorter flowering periods and abnormal flowers^3^. Understanding the heat-resistant mechanism of *Cf*T is a challenging way considering the susceptibility of this species to heat, but could represent a promising new method to get more insights about this valuable and social species, which may help breeders in the future to improve its adaptability to severe environmental conditions such heat. Presently, the molecular mechanism of heat resistance in *Cf*T is poorly understood.

Transcriptome analysis is an effective and widely used technique for exploring genes associated with heat resistance. It has been reported that in switchgrass, a transcriptome analysis had identified 2000 up-regulated and 2809 down-regulated genes under long-term heat stress^4^. In lotus, a transcriptome analysis had revealed the up-regulation of small heat-shock proteins (sHsps) and genes related to cell morphogenesis following heat-shock stress^5^. High temperature stress inhibits the synthesis of normal proteins in plants and increases the synthesis of Hsps^6^. Some studies demonstrated that Hsps function as molecular chaperones under normal conditions^7^. In this regard, they help in nascent peptide folding, assembly, transport and membrane transport under stress conditions. They are also involved in the degradation of misfolded proteins. Hsps repair also aggregated protein to restore their normal functions under stress^8^.

Considering its high ornamental value, importance in landscapes and floriculture, but its high susceptibility to heat stress, clematis (sometime we have to mention clematis instead of *Cf*T) deserves to be more studied to improve its adaptability to severe environmental conditions and eventually to create new varieties to enrich our wealth from this economic and social species^3^. It is recommended herein to focus on the molecular mechanisms adjustment in response to heat stress to detect any potential gene that could modulate the thermotolerance capacity for this species. Therefore, we obtained two lines of clematis with contrasting heat tolerance ability, by screening 300 clematis natural germplasm, and we investigated the physiological and biochemical response. Proteomic screening was used for comparison to our transcriptomic data (RNA-seq), and further validated by qRT-PCR.

The aim of this work is to unravel key genes and/or proteins responsible for the thermotolerance process in *Cf*T using two lines (PS and SG) characterized by their differential capacities to cope up with heat stress. The potential candidate genes and/or proteins that could be identified to be responsible for the acquired thermotolerance in *Cf*T species might be used for the future breeding program to create new lines highly resistant to severe environmental conditions and improve thereby the thermotolerance capacities of the sensitive lines.

## Material and Methods

### Experimental set-up

We have chosen two lines of Clematis (*Clematis florida* Thun, *Cf*T), with contrasting thermotolerance ability from preliminary experiments on evaluation of heat resistance in 300 *Cf*T natural germplasm. The two *Cf*T lines are: Polish Spirit, PS (heat resistance, HR) and Stolwijk Gold, SG (heat sensitive, HS). For heat stress treatments, 3-year old plants of *Cf*T were subjected to a growth chamber (Yiheng Tech. Co. Ltd, China) with 38 °C for 3 h. The air humidity was maintained at around 65%. The middle section of the leaf was subsequently sampled for following experiments, including leaf relative conductivity (LRC), relative water content (RWC), soluble protein, proline and MDA contents.

### Measurements of LRC and RWC

Leaf discs (0.2 g) with diameter 1 cm were placed into a 20 mL tube containing 10 ml distilled water. Then vacuum infiltration was applied for 15 min using specialized machine, then samples were kept for 1 h at room temperature. The conductivity of the water in the tube was determined using a DSS-11 conductivity meter. The tube was then placed in a boiling water bath for 10 min, cooled down to room temperature (23 °C), and the conductivity was measured again. Meanwhile, conductivity in the distilled water was taken as the control. The leaf relative conductivity (LRC) was calculated as the ratio of the difference between conductivities after 1 h versus the control to the difference between conductivity after boiling versus the control. Other leaf discs (0.2 g) were placed in oven at 105 °C for 30 min, and transferred to 80 °C until constant weight, then cooled down to room temperature. Relative water content: RWC = (Fresh weight - Dry weight)/ Fresh weight.

### Determinations of malondialdehyde and soluble protein contents

We chose to assess the extent of lipid peroxidation in leaves because peroxidase is the known as an abiotic stress marker, and accordingly, we expect that the lipid peroxidation would show the big changes. Therefore, we determined the amount of malondialdehyde (MDA) using high performance liquid chromatography (HPLC) as described earlier^9^. The proteins amounts were quantified according to the method^10^. In addition, to investigate the effects of heat stress on ROS scavenging system, one key antioxidant enzymes, superoxide dismutase (SOD, EC 1.15.1.1) was selected for enzymatic assessment based on the reduction of nitroblue tetrazolium (NBT) at 560 nm as described previously^11^. A weight of ~1 g leaf tissue was grounded in liquid nitrogen and extracted in a 5 ml pre-cooled extraction buffer containing 50 mM Tris–HCl pH 7.5 with 1% polyvinylpyrrolidone (PVP). The homogenate was then centrifuged at 20,000 *g* (4 °C, 20 min) and the supernatant was directly used for the enzyme assays. The reaction mixture contains 12.48 μM riboflavin, 13 mM methionine and 75 μM NBT in 0.1 M phosphate buffer pH 7.8 and 50 μl crude enzyme extracts in a total volume of 2.5 ml.

### Evaluations of proline content

The method was applied for the determination of soluble proline content^12^. Fresh leaf tissue (0.5 g) was extracted in 10 ml of 3% sulfosalicylic acid (SSA). Two ml of crude extract was mixed with 2 ml of acid ninhydrin and 2 ml of glacial acetic acid. Then mixture was incubated at 95°C for 60 min. After cooling at room temperature, 4 ml of toluene was added, solution was then vortexed and optical density (OD) of the absorbance of chromophore layer at 520 nm was read with spectrophotometer (IRMECO U2020).

### RNA extraction and library preparation for transcriptome analysis

Three-year old plants from both *Cf*T lines PS and SG either untreated or heat-treated as mentioned above were used for RNAseq and proteomics analysis. The middle section of the leaf was sampled, and total RNAs were extracted by using Trizol reagent according to manufacturer’s instructions (Invitrogen, Carlsbad, CA). The total RNA concentration was quantified with UV spectrophotometer, and the quality was checked by electrophoresis in 1% agarose gel. Equal volumes of RNA from each of the three performed replications for either heat-stress treatment or control were pooled. Six independent paired-end libraries were subjected to RNAseq analysis by second generation sequencing (SGS) technology. Paired-end libraries with average insert lengths of ~200 bp were synthesized by using a Genomic Sample Prep Kit (Illumina, San Diego, CA) according to the manufacturer’s instructions. Prior cluster generation, library concentration and size were assayed using an Agilent DNA 1000 Kit (Agilent, Palo Alto, CA) on a 2100 Bioanalyzer. Libraries were sequenced on an Illumina HiSeq 2500 instrument by a customer sequencing service (Novogene, Beijing, China). The raw data are available in the national center for biotechnology information sequence read archive (www.ncbi.nlm.nih.gov/Trace/sra) under accession number SRP108804.

### Sequencing, de novo assembly and functional annotation

The raw reads were filtered and cleaned by removing low quality raw reads and adaptor sequences. Clean reads were assembled into non-redundant transcripts by using Trinity, which has been developed specifically for the de novo assembly of transcriptomes using short reads^13^. Short sequences (<200 bp) were removed to improve sequencing and assembly quality. The resulting sequences were used in BLAST algorithm-based searches and annotations against NCBI (www.ncbi.nlm.nih.gov/), Kyoto encyclopedia of gene and genomes (KEGG) (www.genome.jp/kegg/kaas/), Clusters of orthologous groups (COG) of proteins (www.ncbi.nlm.nih.gov/COG/) databases, and the manually annotated and curated protein sequence database Swiss-Prot (www.expasy.ch/sport) using an *E*-value cut-off of 10^−5^. The functional annotation of gene ontology (GO) terms was analyzed by using Blast2go software (www.blast2go.com/).

### Protein samples preparation for proteomics

To further understand the global protein expression levels of CfT in response to heat stress, we performed a proteomic analysis using leaf tissues subjected to 37°C heat stress for 3 h obtained from 3-year old plants grown in a growth chamber as mentioned above. The leaf total protein was extracted using cold acetone method as proposed elsewhere^14^. Total protein was extracted from heat treated leaves by using a SDS lysis buffer (2 M thiourea and 1% SDS) with a protease inhibitor. Protein concentrations were detected with a BCA Protein Assay Kit (Pierce, Thermo, USA). Protein digestion and peptide labeling were performed by Duplex TMT. Then, ~50 μg of acetone lyophilized protein pellet of each sample was dissolved with 50 μL of 100 mM triethylammonium bicarbonate (TEAB) as reported previously^15^. Thereafter, pellet was sufficiently suspended and 1.25 μL trypsin per 50 μg protein was added, then kept at 37°C overnight. A TMT-double-stranded isotope labeling reagent set (90063) containing TMT 6–126 and TMT 6–127 labeling reagent vials was dissolved in 40 μL anhydrous acetonitrile for 5 min with gentle shaking. Tubes containing samples were slightly centrifuged (1000 g, 1 min). After tagging for 2 h at room temperature, hydroxylamine was added to react for 15 min at 30 °C. Subsequently, 4 μL 5% hydroxylamine was added to each sample and incubated altogether for 15 min to stop the reactions. The samples were combined in equal amounts and then used for LC-MS/MS analysis as described in the following section.

### LC-MS/MS and data analysis

The mixed samples were fractionated into fractions by ACQUITY Ultra Performance Liquid Chromatography (Waters, USA) with ACQUITY UPLC BEH C_18_ Column (1.7 μm, 2.1 mm × 150 mm, Waters, USA) to increase proteomic depth. Labeled peptides were analyzed by online nano flow liquid chromatography tandem mass spectrometry performed on an 9RKFSG2_NCS-3500R system (Thermo, USA) connected to a Q Exactive Plus quadrupole orbitrap mass spectrometer (Thermo, USA) through a nanoelectrospray ion source. The Q Exactive Plus was operated in the data-dependent acquisition mode (DDA) to automatically switch between full scan MS and MS/MS acquisition. The survey of full scan MS spectra (m/z 350–1300) was acquired in the Orbitrap with 70,000 resolutions. The automatic gain control (AGC) target at 3e^6^ and the maximum fill time was 20 ms. Then the top 20 most intense precursor ions were selected into collision cell for fragmentation by higher-energy collision dissociation (HCD). The MS/MS resolution was set at 35,000 (at m/z 100), the automatic gain control (AGC) target at 1e^5^, the maximum fill time at 50 ms, and dynamic exclusion was 18 seconds. The RAW data files were analyzed using ProteomeDiscoverer (Thermo Scientific, Version 2.2) against *Clematis chinensis* database (http://asia.ensembl.org/Mus_musculus/Info/Index). For both peptides and the MS/MS fragments, the threshold was specified to be ± 0.05 Dalton. Proteins are identified based on the criteria for at least two peptides should match. The mass spectrometry proteomics data has been deposited to the ProteomeXchange Consortium via the PRIDE^16^ partner repository with the dataset identifier PXD018192.

### Identification of differentially expressed genes (DEGs)

We used HTSeq v0.5.4p3 to count the read numbers that are mapped for each gene, then calculated the reads per kilobase per million (RPKM) mapped reads of individual gene based on its lengths and read counts. The resulting adjusted *P-*values, according to the Benjamini and Hoch-berg’s approach, were used to monitor the false discovery rate (FDR). Genes with adjusted *P-*values < 0.01 were considered as “differentially expressed.”

### GO and KEGG enrichment analysis of DEGs

A GO enrichment analysis of DEGs was implemented by mean the GOseq R package, correcting for gene length bias^17^. GO terms with corrected *P-*values < 0.01 were considered significantly enriched by DEGs. For KEGG pathways analysis, we used KOBAS software to determine the statistical enrichment of DEGs^18–20^.

### Quantitative real-time-polymerase chain reaction

Total RNA extraction was same as described above for library construction. Specifically, in this section RNA was treated with DNase I to avoid DNA contamination. The DNase I (RNase-free) (E.C. 3.1.21.1) functions by hydrolyzing phosphodiester linkages, producing mono and oligonucleotides with a 5′-phosphate and a 3′-hydroxyl group according to manufacturer’s instructions (Ambion DNase I, AM2224). Then, 1 mg of RNA was reverse transcribed using Superscript II reverse transcriptase (Invitrogen) with an oligo (dT) 15 primer according to the manufacturer’s instructions (Tiangen Biotech, Beijing, China). Primers were designed by using Primer Premier 5 database (www.premierbiosoft.com/primerdesign/) (Table S1). qRT-PCR experiments were conducted by using SYBR green real master mix (Roche Applied Science, Mannheim, Germany). Reactions were carried out in an ABI 7500 real-time PCR system (Applied Biosystems, Foster City, CA). The actin gene from *Clematis* was amplified as an internal control for data normalization. One PCR reaction volume of 25 μL contains 12.5 μL SYBR Green PCR Master Mix, 9.5 μL deionized H_2_O, 1 μL primers (F + R), and 2 μL cDNA. Amplification reactions were initiated with a pre-denaturing step at 95 °C for 10 min followed by 40 cycles of denaturing at 95 °C for 10 s, annealing at 60 °C for 35 s, then extension at 72 °C for 35 s. Relative expression of gene against housekeeping gene, Actin, was calculated as: 2^−ΔΔCT^ (Δ^CT^ = CT, gene of interest^−CT^, Actin), as described previously^21^. Three complete biological and technical replicates were performed.

## Results

### Physiological profiling in both CfT lines in response to heat stress

**Figure 1:**
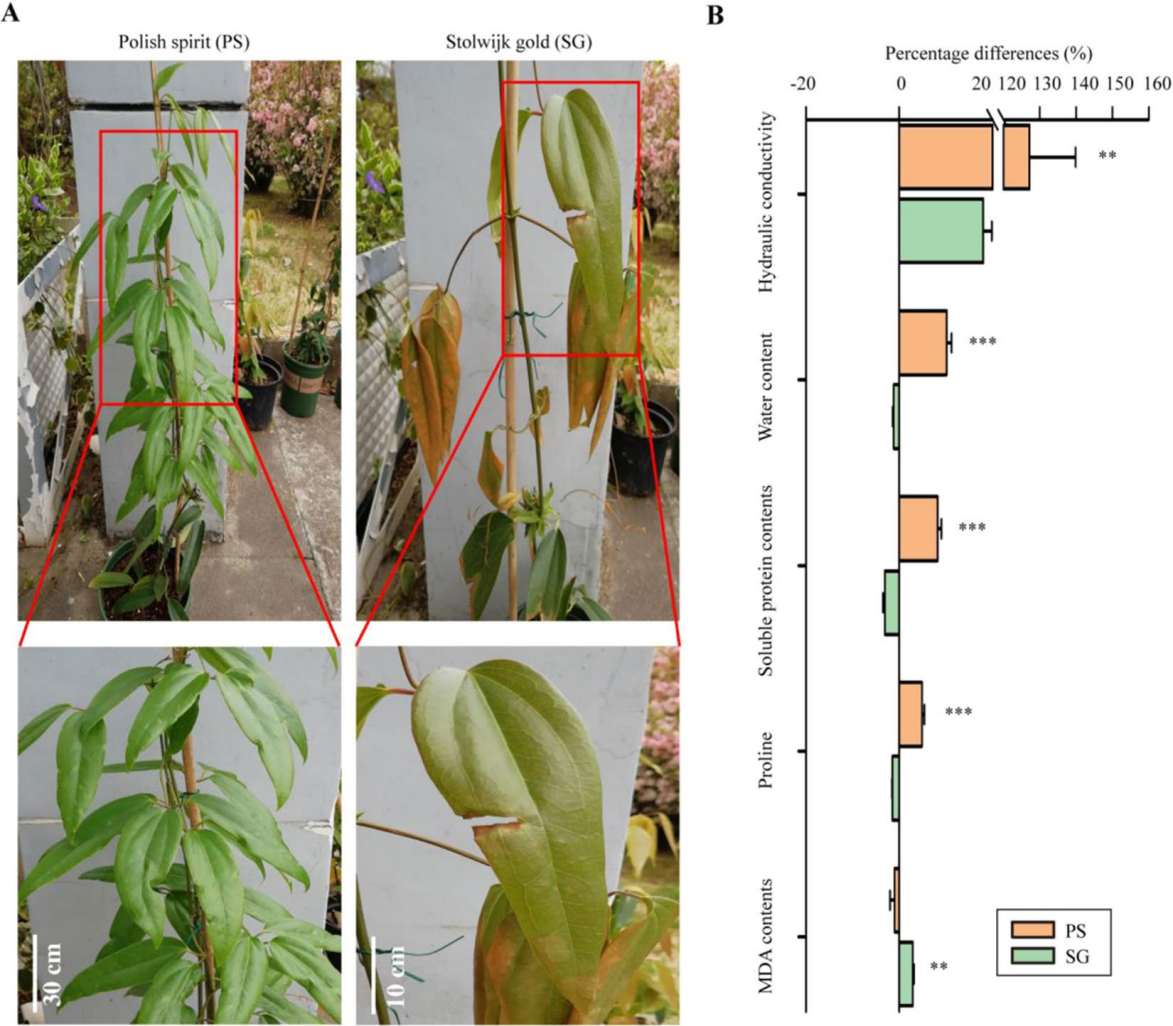
Response of morphological and physiological traits to heat stress in two *Cf*T lines (PS and SG). (**A**) Imaging of PS and SG exposed to heat stress treatments. (**B**) Comparison on physiological traits in two *Cf*T lines. The values show the relative percentage change for each trait in response to heat stress. PS and SG represent Polish Spirit and Stolwijk Gold, respectively. Symbol “*”, “**”, and “***” represent significant levels at *P* < 0.05, 0.01, and 0.001, respectively, based on student-*t* test. Each bar data is the average of 6 replicates ±SE.

In this study, we first determined the morphological and physiological profiling related to heat stress (Fig. 1 and Table S2). The growth and developments were dramatically inhibited by heat stress in *Cf*T heat sensitive line, Stolwijk Gold (SG) rather than *Cf*T heat sensitive line, Stolwijk Gold (SG) (Fig. 1A). Leaf relative hydraulic conductivity (LRC), known also relative hydraulic conductivity (RHC), is tightly related to the integrity of cellular membrane. Our results show an increase in this parameter (RHC) following exposure to heat stress. In particular, SG displays 127% higher RHC due to heat compared to control, while only 18% increase was observed PS (Table S2). In terms of antioxidant signaling pathways, heat stress induced 10%, 8%, and 5% increase in RWC (relative water content), soluble protein amount and proline content in PS, respectively (Fig. 1). While heat induced a decrease by 1%, 3%, and 1% in RWC, soluble protein amount and proline content in SG, respectively (Fig. 1 and Table S2). Heat induced as well a significant decrease by 1% in MDA content for PS; however, it caused its (MDA) increase by 3% for SG (Fig. 1 and Table S2). In addition, heat induced 1.3-fold increase in SOD activity of PS, while only 0.9% increase in SG, relative to the changes in each line under normal condition (Table 1). Altogether, the above mentioned arguments reveal that *Cf*T PS line shows higher heat-resistance than SG one.

**Table 1:** SOD activities of two *Cf*T lines (PS and SG) exposed to heat stress treatments.

*Cf*T lines	Control (%/g. FW)	Heat stress (%/g. FW)	Differences (%)
PS	22.24 ± 0.72	50.96 ± 0.56	129
SG	29.66 ± 1.12	29.93 ± 1.03	0.91

### Integrative analysis of transcriptomes and proteomics in both CfT lines

**Figure 2:**
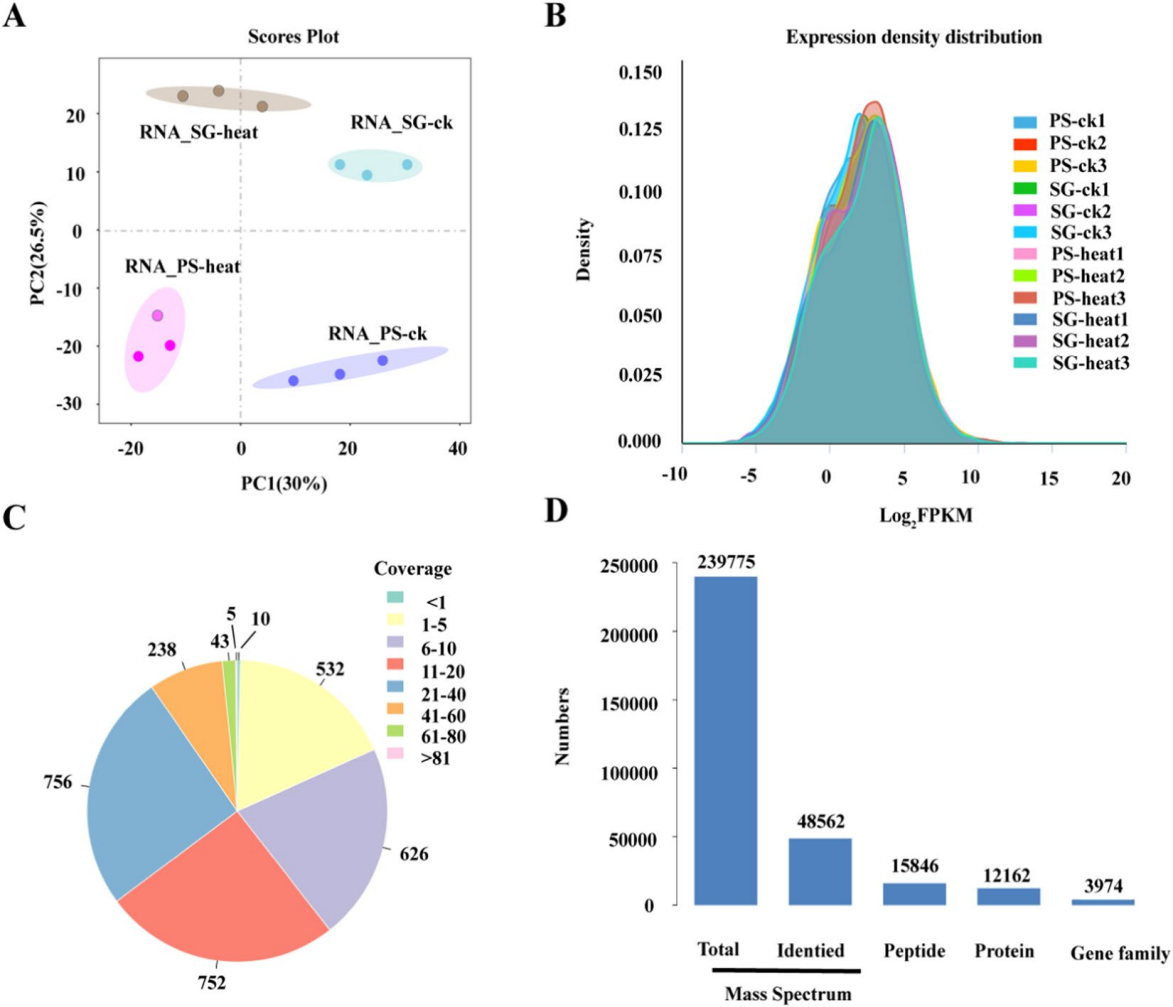
Quality control of proteomics and RNAseq data in two *Cf*T lines (PS and SG) known by their differential thermotolerance performance. (**A**) Principal components analysis (PAC) on RNAseq in PS and SG under control and heat stress conditions. PS and SG are heat tolerance (HR) and heat sensitive (HS), respectively. For transcriptomes analysis, three replicates were used for either control (ck) or heat-stressed (heat) PS and SG. (**B**) Histogram represents the distribution of global gene expression density in PS and SG under both control and heat stress conditions. (**C**) Protein coverage and the corresponding peptides numbers. (**D**) Numbers of peptides and protein identified by LC-MS/MS. For proteomics, we have performed three independent replicates for each line and each condition.

To better understand the clematis response to heat stress at the molecular level, we performed both transcriptomes and proteomics analysis in two *Cf*T lines. In this regards, the principal component analysis (PCA) obtained from transcriptome study showed a distinct separation between control and heat-treated plants for the same line or between lines themselves (Fig. 2A). This is also confirmed for the proteomic analysis (Fig. S2A). The PCA calculated from the proteomic screening follows similar segregation between PS control and SG heat-treated (Fig. 2A) to that recorded for transcriptome. The expression density in different ranges of FPKM across all treatments yields normal distribution (Fig. 2B). The majority of proteins coverage is 6~40 for proteomics (Fig. 2C). In terms of proteomics, the identified mass spectrum account for 20% in total mass spectrum, and among these, 32% and 25% were identified for peptides and protein, respectively (Fig. 2D).

The changes of gene expression could lead to an alteration in the abundance of proteins in response to an abiotic stressful condition. We observed strong correlation regarding the DEGs in both *Cf*T lines (PS and SG) (Fig. S1A). We identified 7294 and 6983 up- and down-regulated DEGs in heat tolerant *Cf*T line (PS), respectively, if compared to the heat-sensitive one, SG (Fig. S1A,B). This reveals that heat stress leads to an important reshuffling in the transcriptome of PS (HR) but not or very less for SG (HS). This is very likely might be explained as a part of stress tolerance mechanisms, which may allow the plant to synthesis of some specific Hsp and the degradation of other unusable normal proteins during the acclimation process to severe stressful conditions. GO and KEGG analysis were performed to identify the potential biological pathways or metabolic process responsible for clarifying the differential thermoltolerance capacities between these two *Cf*T lines (Fig. S1C,D). The gene ontology (GO) analysis shows that the regulation of nucleobase-containing compound metabolic process, protein serine/threonine phosphatase complex and cation antiporter activity were enriched in cluster of biological process, molecular function and cellular components, respectively (Fig. S1C). For Kyoto Encyclopedia of Genes and Genomes (KEGG) analysis, our findings displayed that plant hormone signal transduction, starch and sucrose metabolism, Glyoxylate and decarboxylase metabolism were significantly enriched in list of DEGs (Fig. S1D).

Regarding the proteomics analysis, we observed a strong correlation between data of both *Cf*T lines. The proteomics data sweeping revealed that 496 and 591 identified proteins were up- and down-regulated, respectively (Fig. S2B,C). Moreover, GO and KEGG analysis were also performed at the proteomics level, to characterize any potential biological pathway or metabolic process that could help understanding the differential thermoltolerance response to heat stress between these two *Cf*T lines (Fig. S3). In this regards, our results showed that antioxidant activity, cellular membrane, and response to stimulus were significantly enriched in cluster of molecular function, cellular components and biological function, respectively (Fig. S3A). In addition, we found that some metabolic pathways were significantly enriched based on KEGG classifications, such as environmental adaptation, amino acid metabolism and signaling transduction (Fig. S3B).

**Figure 3:**
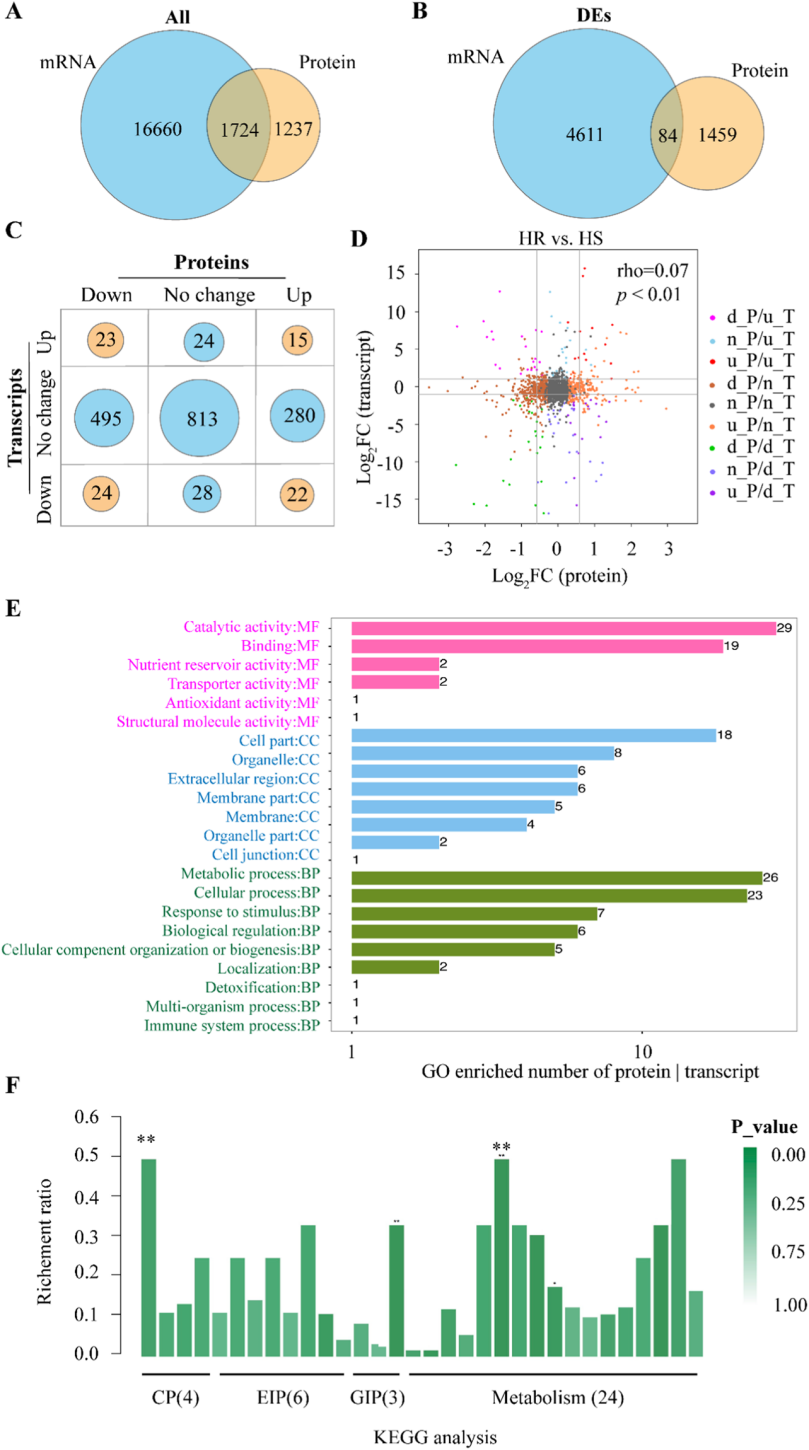
Integrative analysis of proteomics and transcriptomes induced following heat stress in two *Cf*T lines (PS and SG). (**A**) Venn diagram represents the overlapped events in all identified proteins and transcripts. (**B**) Venn diagram represents the overlapped events in only differentially expressed proteins (DEPs) and transcripts. (**C**) Bubble chart represents the numbers of genes and proteins with different expression pattern induced by heat stress. The expression patterns include down regulation, up-regulation and no significant effects due to heat stress in HR over HS lines of *Cf*T. (**D**) Comparison on transcripts and proteins with down/up/no significant changes. (**E**) GO enrichment analysis on both DEPs and transcripts. (**F**) KEGG analysis on both DEPs and transcripts. For the bottom panel (panel F), the abbreviations of different pathways were as listed below: CP (cellular processes): biofilm formation, endocytosis, peroxisome, tight junction; EIP (Environmental information processing): AMPK signaling pathway, FoxO signaling pathway, MAPK signaling pathway, MAPK signaling pathway-fly, MAPK signaling pathway-plant, plant ABA signal transduction; GIP (Genetic information processing): protein processing in endoplasmic reticulum, ribosome, spliceosome; M (metabolism): glutathione metabolism, glyoxylate and dicarboxylate metabolism, carbon metabolism, biosynthesis of amino acids, biosynthesis of antibiotics, carbon fixation in photosynthetic organisms, chloroalkane and chloroalkene degradation, cyanoamino acid metabolism, degradation of aromatic compounds, drug metabolism-cytochrome P450, drug metabolism-other enzymes, fatty acid degradation, galactose metabolism, isoquinoline alkaloid biosynthesis, metabolism of xenobiotics by cytochrome P450, naphthalene degradation, nitrogen metabolism, oxidative phosphorylation, pentose and glucoronate inter-conversions, phenylpropanoid biosynthesis, photosynthesis, retinol metabolism, starch and sucrose metabolism, tyrosine metabolism; the numbers of genes involved in each pathway were indicated in bracket.

Furthermore, we performed an integrative analysis on proteomics and transcriptomes on two *Cf*T lines (PS and SG) exposed to short-term heat (3 h at 38 °C). Thus, 1724 overlapped genes were identified based on combined transcriptomes and proteomics analyses across two *Cf*T lines (Fig. 3A). We further identified, through this integrative analysis, 84 differentially expressed genes (DEGs) between PS and SG *Cf*T lines in response to heat stress (Fig. 3B). As depicted in the bubble chart of Fig. 3C, among the 84 identified DEGs, 15 were up-regulated and 24 down regulated in PS (HR) line of *Cf*T following heat stress (Fig. 3C). Altogether, the 15 upregulated gene and proteins were listed in Table 2. These 84 DEGs were included in the plot of Fig. 3D. GO analysis in the list of genes and proteins differentially expressed suggests that the top single enriched pathway for molecular function (MF), cell components (CC), and biological pathways (BP) is catalytic activity, cell part and metabolic process, respectively (Fig. 3E). KEGG analysis shows that metabolic pathways such as peroxisome, biosynthesis of amino acids, carbon fixation in photosynthetic organisms, carbon metabolism, glyoxylate and dicarboxylate metabolism, oxidative phosphorylation, photosynthesis were significantly enriched (Fig. 3F).

**Table 2:** Up-regulated 15 genes in *Cf*T PS line in both transcriptomes and proteomics analyses.

Protein	Transcript	Abbre	Description
cdsTRINITY_DN115825_c1_g2_m58122	ORTHOMCL14236	GLUST	Glutathione-S-transferase tau 1 (*Hevea brasiliensis*)
cdsTRINITY_DN117586_c0_g2_m50305	ORTHOMCL16012	GO1	Gglycolate oxidase1, chloroplastic-like (*Fragaria vesca* subsp Vesca)
cdsTRINITY_DN108848_c1_g2_m66744	ORTHOMCL7824	RPE3	Ribulose-phosphate 3-epimerase, cytoplasmic isoform (*Malus domestica*)
cdsTRINITY_DN111615_c0_g1_m847	ORTHOMCL10183	R5PI	Ribose-5-phosphate isomerase 3, chloroplastic (*Cucumis melo*)
cdsTRINITY_DN108758_c3_g1_m45874	ORTHOMCL7756	rbcS	Ribusco small subunit (*Panax ginseng*)
cdsTRINITY_DN117844_c1_g1_m47440	ORTHOMCL16305	POD4	Peroxidase 4 (*Vitis vinifera*)
cdsTRINITY_DN113183_c1_g1_m25469	ORTHOMCL11612	HSP18	18 kDa class II heat shock protein-like (*Malus domestica*)
cdsTRINITY_DN105472_c0_g1_m18350	ORTHOMCL5475	HSP70	Heat shock cognate 70 kDa protein 2-like (*Cucumis melo*)
cdsTRINITY_DN94901_c0_g1_m31462	ORTHOMCL18094	PRP1	Putative pathogenesis-related protein 1 (Vitis hybrid cultivar)
cdsTRINITY_DN102906_c0_g1_m54008	ORTHOMCL4000	EP1-like	Epidermis-specific secreted glycoprotein EP1-like (*Solanum tuberosum*)
cdsTRINITY_DN111577_c2_g1_m15214	ORTHOMCL10140	Pru2A	Putative allergen Pru du 201 A (*Prunus dulcis* x *Prunus persica*)
cdsTRINITY_DN106744_c0_g1_m25289	ORTHOMCL6285	tNFTR	Tyramine N-feruloyltransferase 4/11-like (*Fragaria vesca* subsp Vesca)
cdsTRINITY_DN115453_c0_g1_m64076	ORTHOMCL13846	EUTSA	Hypothetical protein EUTSA_v10008067mg (*Eutrema salsugineum*)
cdsTRINITY_DN107522_c0_g1_m23602	ORTHOMCL6831	21 kDa	21 kDa protein-like (*Fragaria vesca* subsp Vesca)
cdsTRINITY_DN111222_c2_g1_m34930	ORTHOMCL9814	RPT1A	Regulatory particle triple-A 1A (*Theobroma cacao*)

For the original data of these 15 genes refer to the bubble chart of Fig. 3C. Abbre. means abbreviation.

**Figure 4:**
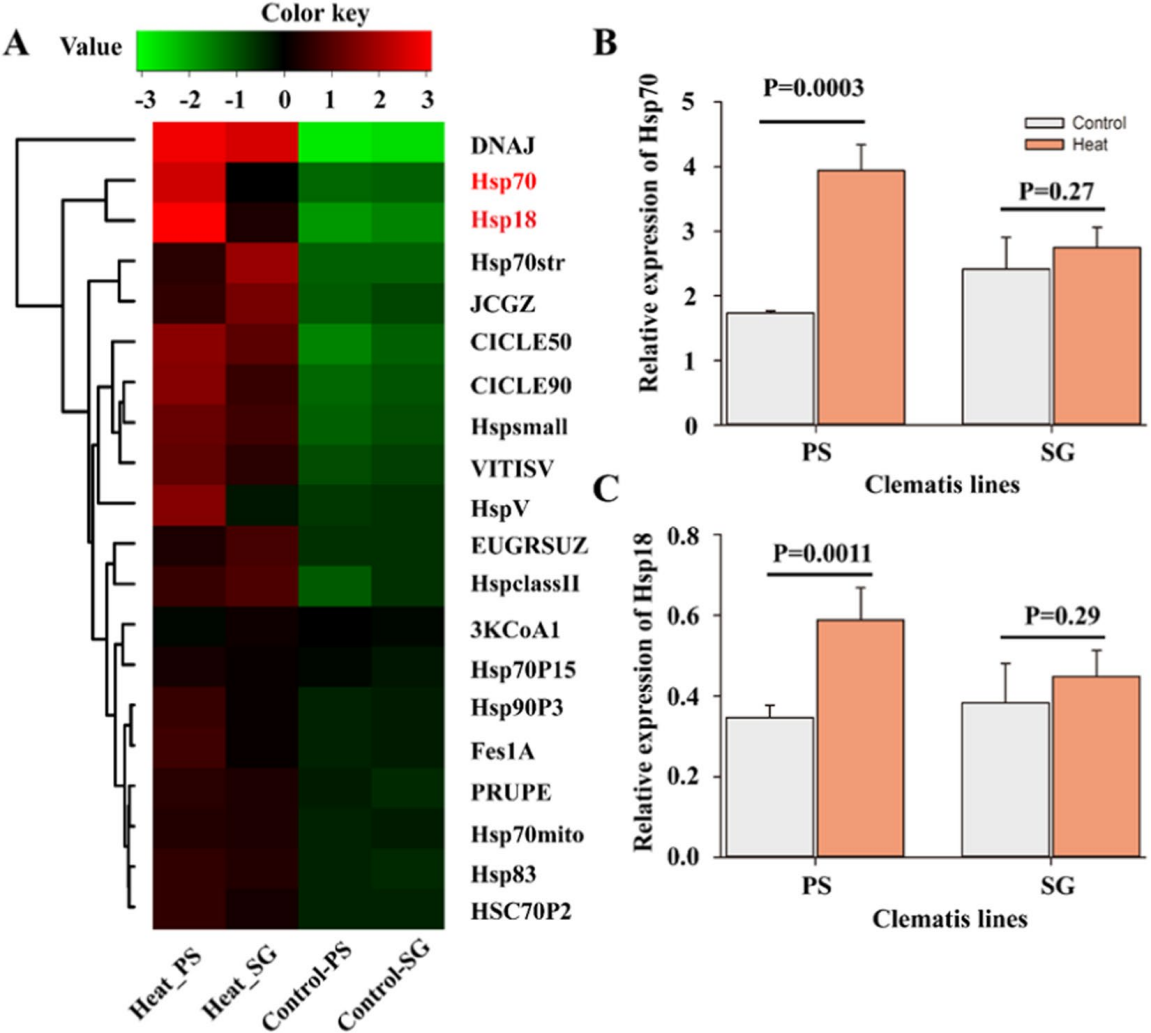
Heatmap displaying the differentially expressed heat shock protein family in transcriptome in two *Cf*T lines (PS and SG) following heat stress treatment. (**A**) Expression of heat shock protein gene family in response to heat stress in PS and SG. The heatmap was generated using the heatmap3 package of the R software (R-3.5.2, www.r-project.org). (**B,C**) Relative expression of two extremely expressed genes between PS and SG based on qPCR experiments. Each bar data is the average of 3 replicates ±SE.

In higher plants, a rapid expression reprogramming of numerous proteins known as heat shock proteins (Hsps) could be considered as heat stress markers and being part of the heat stress tolerance mechanism. We hence compared all annotated Hsps consisting of 20 genes selected in this study (Fig. 4A and Table S3), with substantially different expression levels between control and heat-treated plants of both *Cf*T lines (PS and SG). Results from transcriptomes data show that Hsp70 and Hsp18 displayed higher expression levels in PS than SG in heat-treated plants (Fig. 4A and Table S3), while no significant difference was observed for both lines without heat stress (Fig. 4B). The expressions of Hsp70 and Hsp18 were further confirmed by qPCR (Fig. 4B). The differences in expressions of Hsp70 and Hsp18 either between heat-stressed *Cf*T lines (PS and SG) or between untreated and heat-treated plants of PS line were very significant (*P* < 0.001) (Fig. 4B,C). These results suggest that Hsp70 and Hsp18 are strongly involved in the thermololerance process and are responsible for the acquired thermolerance in both *Cf*T lines (PS and SG) but to a higher extent for PS.

**Figure 5:**
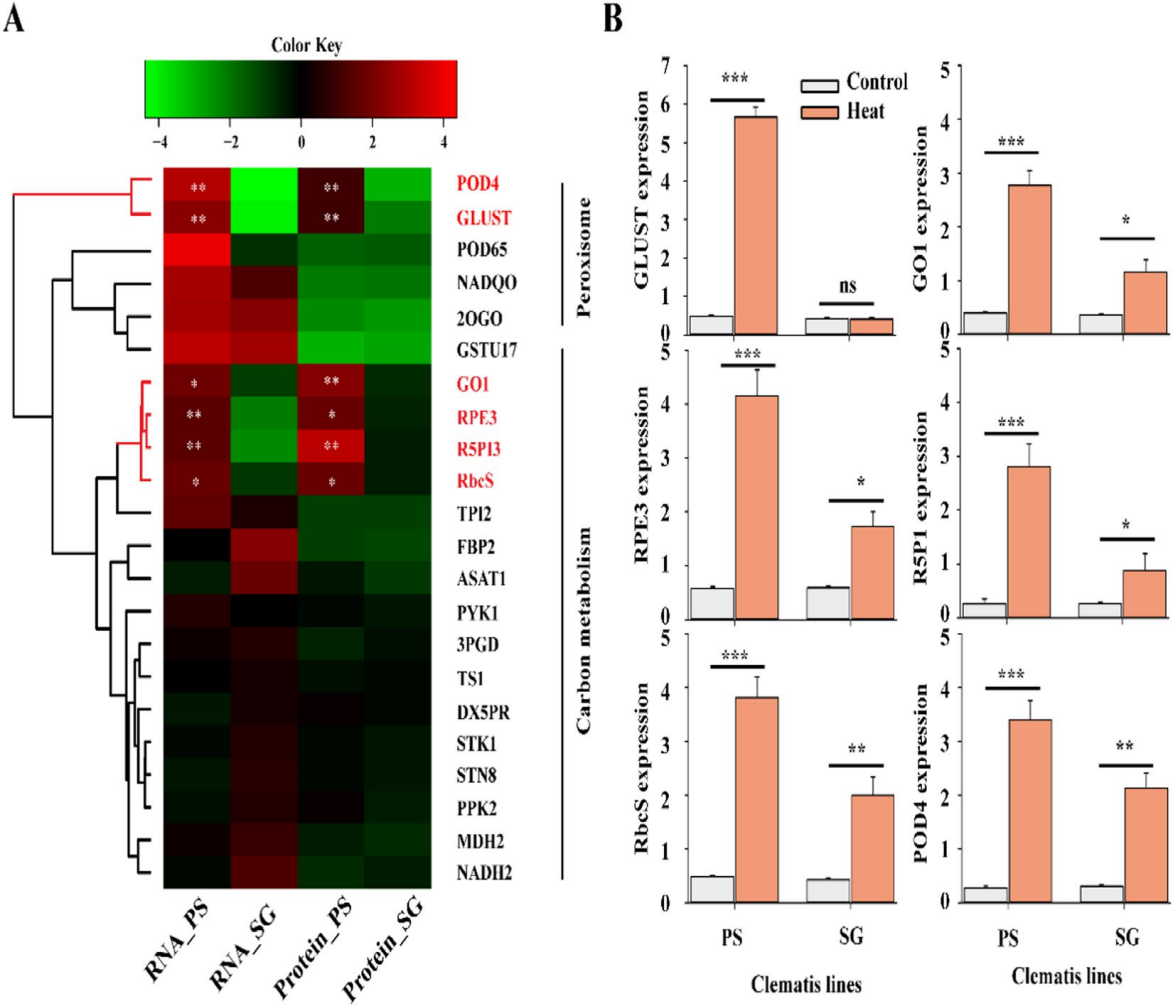
Determination of transcripts and proteins involved in the enriched metabolic pathways. (**A**) Heatmap represents the relative abundance of genes of proteins under heat shock treatments in peroxisome and carbon metabolism pathways. The heatmap was generated using the heatmap3 package of the R software (R-3.5.2, www.r-project.org). (**B**) Gene expression level of six significantly abundant genes in transcriptomes and proteomics analyses in PS line based on qPCR experiments. Each bar data is the average of 3 replicates ±SE.

Based on the key enriched metabolic pathways depicted in Fig. 3F, we further analyzed the list of DEGs in peroxisome and carbon metabolism pathways, which consists of 5 and 17 genes, respectively (Fig. 5A and Tables S4 and 5). In particular, two genes which are peroxidase 4 (*POD4*) and glutathione-S-transferase tau 1 (*GLUST*) belong to peroxisome pathway and four other genes (Fig. 5A), glycolate oxidase1 (*GO1*), ribulose-phosphate 3-epimerase (*RPE3*), ribose-5-phosphate isomerase 3 (R5PI3) and RbcS (Fig. 5A and Tables S4 and 5) from the carbon metabolism are significantly highly abundant in PS than in SG in both transcriptomes and proteomics data (Fig. 5A).

To gain more insights on these 6 genes regarding their expression levels between *Cf*T lines (PS and SG), under either control or heat stress conditions, we performed qRT-PCR analysis. Therefore, the qPCR results reflect no significant difference in the expression levels between both *Cf*T lines for these six genes (*GLUST*, *GO1*, *RPE3*, *P5PI3*, *RbcS* and *POD4*). However, their expression levels were enhanced by 2∼4 times in PS if compared to SG after exposure to heat stress (Fig. 5B and Tables S4 and 5).

**Figure 6:**
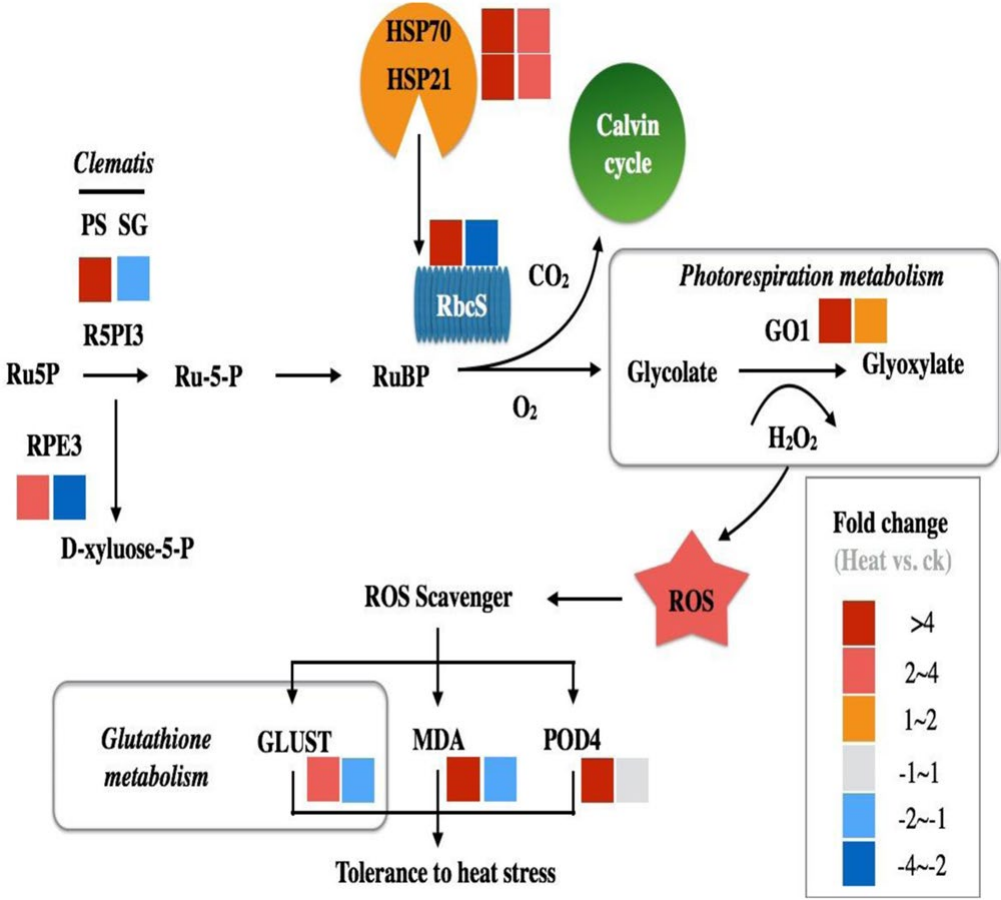
Representative schematic model displaying the effect of heat stress on photosynthetic carbon metabolism and ROS scavenging system for both *Cf*T lines (PS and SG). Cells in gradient colors represent the relative ratio of proteins or values of physiological parameters in PS and SG *Cf*T lines exposed to heat stress. Regarding the model, most of pathways (carbon metabolism, photorespiration and ROS scavenging system) operate in synergy (fold of change was mostly higher than 4 for all sites), which potentially reflects the high protection and resistance of PS line to heat stress. However, for SG line, except the photorespiration metabolism (fold of change between 1~2), all the other sites were down regulated and this very likely could be the reason of the high vulnerability of SG to heat.

With help of the DEGs identified above, we propose a representative schematic model to summarize the mechanism of heat tolerance in *Cf*T (Fig. 6). We highlighted as well the importance of Hsps, photorespiratory metabolism, ROS scavenging system in heat resistance in clematis. Besides, we conclude that the enhanced transcripts and proteins of six genes (*GLUST*, *GO1*, *RPE3*, *P5PI3*, *RbcS* and *POD4*) together with two crucial stress markers proteins (*Hsp18* and *Hsp70*) represent the key events for the acquired thermotolerance in PS *Cf*T line. Thereby, we approximately have drawn a general view and dissect the differential thermotolerance capacities between PS and SG. However, understanding concretely and more deeply mechanisms of tolerance at the nucleotide level needs more fine and accurate molecular experiments.

## Discussion

*Cf*T is a valuable species by its high ornamental and medicinal importance^2^. It is extensively cultivated in landscapes and floriculture. Thereby, it may possess a huge impact on the economic and social sectors. It is a cold resistant but heat sensitive plant^3^. However, the molecular mechanism of its response to heat stress remains less reported and needs to be more investigated^3^. In this study, we focus on the physiological and omic-levels in the response in two *Cf*T lines (PS and SG) to short-term heat stress. Overall, our preliminary test identified two lines with differential response to heat stress through screening 300 *Cf*T natural germplasm. Notably, PS is a heat-resistant (HR) line and SG is a heat-sensitive one. Following heat stress, the genes in the enriched metabolic pathways based on transcriptomes and proteomics were identified and further amplified and quantified using qRT-PCR. The molecular relevance of these pathways in the thermotolerance process in *Cf*T will be obviously discussed below.

### Benefits of water relation against heat stress in heat-tolerant clematis lines

High temperature is one of the main environmental factors that affects plant life cycle activities. In heat sensitive species, heat stress could enhance leaf temperature and thus inhibits the activities of photosynthetic enzymes^22^. However, heat-tolerant plants possess the abilities to prevent the alteration of cellular process by heat stress through regulating the leaf water status^23^. One strategy attributable to the pressure of transpiration rates and leads to the acceleration in the hydraulic conductivity, which ultimately ensures cooling the temperature of leaf surface, as observed in many species via thermal imaging^23^. Vigorous root system may constitute another adaptive strategy, which supply sufficient water to canopy aboveground evaporation and mitigate the damage to cell membrane system due to drought stress^24^. We observed higher hydraulic conductivity and leaf water content in heat-tolerant *Cf*T line (PS) than heat-sensitive one (SG), suggesting that the ability of heat tolerance is dependent on the efficient regulation of water status in *Cf*T species as observed in other species^23^.

### Heat shock proteins and clematis heat tolerance

As an adaptive strategy, plants trigger a series of mechanism, such as MPK/AMPK signaling pathway as identified in the enriched cellular process in the accumulated proteins list of HR *Cf*T (PS) line compared to HS one (Fig. 3). This signaling pathway is able to stimulate the expression of heat shock protein genes through phosphorylation, such as Hsp27, a member of small Hsp family^25^. Hsps are commonly induced in response to heat stress but could occur also following exposure of plant to increased salinity levels and other kinds of stresses such as drought^26^. Hsps typically act as molecular chaperones regulating the folding, accumulation, localization and degradation of normal proteins^7^. Based on the diversity of their N-terminal domains, function and molecular weight, these Hsps are classified into five groups: Hsp100, Hsp90, Hsp70, Hsp60 and sHsps (small heat-shock proteins, such as Hsp18). The enhanced capacity of heat tolerance is certainly accompanied by the synthesis of Hsps. This is in line with our finding regarding the HR *Cf*T line (PS), where we found that the higher thermotolerance capacity was concomitant to an increase in the expression of some Hsps and the appearance of enhanced expression of previously identified Hsps.

Hsp70, an important member of Hsps gene family, has an essential function in preventing the aggregation and/or assisting refolding of non-native proteins under both normal and stressful conditions, ensuring the maintaining of correct protein structure. Hsp70 also participates in the degradation of dysfunctional or poorly folded proteins and interacts with intercellular proteins and signaling molecules^27^. It has been reported that Hsp70 is able to recognize the domain within the Rubisco small subunit transit peptide protein^28^. This suggests that the synthesis of Hsp70 is involved in the modulation of the photosynthetic carbon metabolism under heat stress conditions as well as under other abiotic stress^29^. In addition to Hsp70, many sHsps are also involved in carbon metabolism, for instance, Hsp21 was found to interact with the plastid nucleoid protein pTAC5 and demonstrated to be necessary for the development of chloroplasts during heat stress in Arabidopsis^30^. In *Oryza sativa*, OsHsp26 plays a major role in the protection of photosystem II during heat stress of 42 °C^31^. In line with the above, we have emphasized in a recently published results performed on *Zea mays* a strong correlation between the sHsps amplification levels and the carbon assimilation rate under sudden heat shock (SHS) stress^29^. Consistently, we demonstrated in the current study an up-regulated of Hsp18 is HR *Cf*T line (PS) but not or very less for the HS one (SG).

### Comprehensive regulation of photosynthetic carbon metabolism under heat stress

Photosynthetic carbon metabolism is the first target for abiotic stress in the photosynthetic apparatus and it represents an efficient stress sensor that firstly detects the exogenous stimulus and responds immediately to stressful conditions compared to other biological process. The key enzyme of the carbon assimilation metabolic pathway is Ribulose-1,5-bisphosphate carboxylase/oxygenase (Rubisco) carboxylation and oxygenation^32^, which may define the running of photosynthesis or photorespiration. Higher ability of Rubisco catalyzing RuBP using CO_2_ as substrates always observed in heat-tolerant species^33^, which requires NADPH and ATP from fixation stage during Calvin cycle. In this regards, amino acids and protein are synthesized at the cost of both energy (ATP) and reducing power (NADPH)^34^. However, photorespiratory metabolism is always coupled with carbon assimilation due to dual-functional features of Rubisco^35^, which leads to H_2_O_2_ production as observed high expression of glycolate oxidase1 (*GO1*) gene and its coding protein, GO1 oxidizing glycolic acid to glyoxylate (Fig. 5A,B). In addition, we found that the amounts of most photosynthetic enzymes were enhanced, such as ribulose-phosphate 3-epimerase (RPE3), ribose-5-phosphate isomerase 3 (R5PI3) and Rubisco small subunits (RbcS) in heat-tolerant clematis (Fig. 5A,B). The accumulation of these proteins could be resulted by the increased total soluble protein amount in heat-tolerant *Cf*T line, PS (Fig. 1). For instance, the content of LHCII accounts for approximately 50% of all proteins in the thylakoid membranes^36^.

### Mechanisms of proline stress protection coupling with ROS scavengers

Reactive Oxygen Species (ROS) is a global expression used to describe a number of reactive molecules and free radicals derived from molecular oxygen (O_2_), which may resulted in a significant damage to cell structures under environmental stress such as heat. The main source of ROS is the accelerated rate of photorespiration^37^, which contributes to more than 70% of the generated hydrogen peroxide, H_2_O_2_^38^. Malondialdehyde (MDA), one of the main products of lipid peroxidation, is an important biomarker reflecting oxidase stress and involved in ROS scavenging. In this study, the lower MDA content in HR *Cf*T line (PS) than that in HS one, SG (Fig. 1) due to less occurrence of membrane lipid peroxidation, which prevents electrons conversion through free radicals from the lipids in cell membranes and cell damage. Previous reports have shown that this stress marker molecule (MDA) is derived from lipid peroxidation in membranes followed by H_2_O_2_ production when plants exposed to heat stress^39,40^.

Considering the combined dataset of transcriptomes and proteomics in this study, we didn’t found strong correlation between two dataset, and only 84 genes with significant differences regarding their expression of gene and protein levels (Fig. 3B). Noticeably, among them, we found some genes are related to ROS scavenging system such as peroxidase. In this work, we noticed a high expression of *POD1* gene and its coding protein (Fig. 5A,B). Glutathione is an antioxidant enzyme in plants, capable of preventing damage to important cellular components that could be caused by oxidative stress and thus by ROS such as free radicals and peroxides, and other forms (lipid peroxides, and heavy metals). We recorded the abundance and accumulation of glutathione-S-transferase (GLUST), catalyzing the conjugation of the reduced form of glutathione to xenobiotic substrates for the purpose of detoxification, in heat-tolerance *Cf*T (Fig. 5A,B). Proline is a compatible osmolite acts as low-molecular weight chaperones and avoids water loss in stress-treated plants^41^. Notably, proline may help stabilizing and protects the enzymes and proteins structures, maintain membrane integrity and scavenges ROS. The accumulation of proline in the HR *Cf*T line (Fig. 1), together with other molecular factors (Hsp70, Hsp18 and POD1), could enhance its tolerance and strengthen the antioxidant activities to detoxify the deleterious elements from the cells.

## Conclusion

We carried out an integrative analysis on proteomics and transcriptomes in two *Cf*T lines (PS and SG) known by their differential thermotolerance capacities. High contents of proline and soluble protein but less MDA content were recorded in *Cf*T HR line (PS). The reverse was true for the HS line (SG), where we observed low proline and soluble protein contents but high MDA level. Furthermore, 15 DAGs were identified to increase their expressions in terms of mRNA and protein levels in *Cf*T HR line (PS), including two heat shock proteins (Hsp18 and Hsp70), and mainly six other genes (*GLUST*, *GO1*, *RPE3*, *P5PI3*, *RbcS* and *POD4)* involved in the carbohydrates metabolism and antioxidant pathways.

## Supplementary information

Supplementary Information.
