# Supplementary material for: Proteome and transcriptome reveal the involvement of heat shock proteins and antioxidant system in thermotolerance of Clematis florida
Source: Sci Rep. 2020 Jun 1;10:8883. doi: 10.1038/s41598-020-65699-2 (PMC7264250; doi:10.1038/s41598-020-65699-2)
Supplement: Supplementary file 1 — Supplementary Information. [file 41598_2020_65699_MOESM1_ESM.docx]

**Supplementary materials**

**Proteome and transcriptome reveal the involvement of heat shock proteins and antioxidant system in thermotolerance of *Clematis florida***

Changhua Jiang^1,#^, Yuke Bi^1,#^, Jianbin Mo^1^, Ruyao Zhang^1^, Mingnan Qu^2^, Shucheng Feng^1,*^, Jemaa Essemine^2,*^

^1^Shanghai Botanical Garden, Shanghai, 200231, China

^2^CAS Center for Excellence in Molecular Plant Sciences, Institute of Plant Physiology and Ecology, Shanghai Institutes for Biological Sciences, Chinese academy of Sciences, Shanghai, 200032, China;

***Corresponding author:**

*Shucheng Feng* ([498842061@qq.com](mailto:498842061@qq.com)); *Jemaa Essemine* (jemaa@picb.ac.cn)

^#^ Authors equally contributed to this work.

Numbers of Figures: 6

Numbers of Tables: 1

Numbers of Supplemental Figures: 3

Numbers of Supplemental Tables: 5

Numbers of Words: 7064

**Author contributions:** Conceptualization, S.F., E.J.; Methodology, M.Q., J.M., R.Z.; Investigation, C.J., Y.B.; Writing, M.Q., E.J.; Funding Acquisition, S.F., M.Q. Resources, S.F., C.J, E.J.; Supervision, S.F., E.J.

**Supplemental Tables**

**Table S1.** Primers list used in this study.

| **Gene ID.** | **Primer name** | **Primer sequences** | **Product size** |
| --- | --- | --- | --- |
| POD4 | POD4-F | CTATGATCTTAGGAGCACTG | 193 |
|  | POD4-R | GCTAGAACTATGGCGAAT |  |
| GLUST | GLUST-F | TATGCTGGTAACAAGTGAT | 122 |
|  | GLUST-R | TGACAACGAAAGTAATGG |  |
| GO1 | GO1-F | TCAGGGAGCATTTAGAGT | 184 |
|  | GO1-R | GGTTCTTATGGGTTGACT |  |
| RPE3 | RPE3-F | AAGCTCTACGCTCCTGAA | 110 |
|  | RPE3-R | TCTGACCCGCATAACAAT |  |
| R5PI3 | R5PI3-F | ATAGTGAACGGAGACCAGC | 170 |
|  | R5PI3-R | CGACTAGCCAAAGGAACA |  |
| RbcS | RbcS-F | GCTAATAACAACATCCCTC | 156 |
|  | RbcS-R | ATTGTAAATTCTGGCTCTG |  |
| HSP70 | HSP70-F | GGATGTCCGCTTCTTCGA | 121 |
|  | HSP70-R | TGCCTTGGCGTCTTGATA |  |
| HSP18 | HSP18-F | CTCTTTCAAGCATCCCAGTC | 106 |
|  | HSP18-R | CATCCTCCACCGTCACCT |  |
| Actin | Actin-F | AGTGGAGTAGACGAAAGTG | 183 |
|  | Actin-R | TGGTGGGATGATAGAAGT |  |

**Table S2**. Comparison on the physiological traits in two Cft (PS and SG) under control and heat stress conditions.

| Parameters | Clematis lines | Control | | Heat stress | |
| --- | --- | --- | --- | --- | --- |
|  |  | Mean | s.e. | Mean | s.e. |
| Hydraulic | Polish Spirit (PS) | 117.62 | 15.89 | 138.84 | 1.44 |
|  | Stolwijk Gold (SG) | 96.63 | 19.56 | 219.49 | 12.84 |
| Relative water | Polish Spirit (PS) | 71.09 | 3.93 | 78.34 | 8.15 |
|  | Stolwijk Gold (SG) | 75.01 | 2.92 | 74.08 | 11.34 |
| Soluble protein | Polish Spirit (PS) | 7.12 | 1.89 | 12.99 | 1.42 |
|  | Stolwijk Gold (SG) | 9.12 | 1.16 | 8.84 | 1.82 |
| Proline | Polish Spirit (PS) | 54.41 | 0.62 | 59.32 | 0.73 |
|  | Stolwijk Gold (SG) | 51.69 | 0.62 | 50.24 | 0.34 |
| MDA | Polish Spirit (PS) | 31.22 | 0.24 | 30.24 | 0.34 |
|  | Stolwijk Gold (SG) | 44.25 | 0.14 | 47.15 | 0.24 |

**Table S3.** Abundance of heat shock protein gene family in two Cft (PS and SG) induced by heat stress treatments based on transcriptomes and proteomics

| **Protein** | **Abbrev.** | **Transcript** | **Log2FC(PS/SG)-Protein** | **Significant** | **Regulate-Protein** | **Log2FC(PS/SG)-Transcript** | **Significant** | **Regulate- Transcript** | **Description** |
| --- | --- | --- | --- | --- | --- | --- | --- | --- | --- |
| **cds.TRINITY_DN104690_c0_g1_m.30385** | **HSPV** | ORTHOMCL4992 | 0.005599082 | no | no change | -4.85 | yes | down | 15.4 kDa class V heat shock protein [Medicago truncatula] |
| **cds.TRINITY_DN117660_c1_g1_m.29640** | **HSP90P3** | ORTHOMCL16086 | -0.358171615 | yes | down | -0.98 | no | down | PREDICTED: hsp70-Hsp90 organizing protein 3 [Phoenix dactylifera] |
| **cds.TRINITY_DN119523_c1_g3_m.6041** | **DNAJ** | ORTHOMCL18190 | -0.095625717 | no | no change | 0.52 | no | up | DNAJ heat shock family protein [Theobroma cacao] |
| **cds.TRINITY_DN113183_c1_g1_m.25469** | **HSP18** | ORTHOMCL11612 | 1.23442 | yes | up | 5.81 | yes | up | 18 kDa class II heat shock protein-like (Malus domestica) |
| **cds.TRINITY_DN105472_c0_g1_m.18350** | **HSP70** | ORTHOMCL5475 | 2.131663623 | yes | up | 4.59 | yes | up | PREDICTED: heat shock cognate 70 kDa protein 2-like [Cucumis melo] |
| **cds.TRINITY_DN119090_c1_g3_m.17017** | **HSP70stro** | ORTHOMCL17685 | 0.434259718 | yes | up | -1.27 | yes | down | PREDICTED: stromal 70 kDa heat shock-related protein, chloroplastic-like [Vitis vinifera] |
| **cds.TRINITY_DN106069_c0_g1_m.29708** | **JCGZ** | ORTHOMCL5867 | 0.903175622 | no | up | 2.88 | yes | up | hypothetical protein JCGZ_05100 [Jatropha curcas] |
| **cds.TRINITY_DN104377_c0_g1_m.49020** | **CICLE50** | ORTHOMCL4803 | 1.402139879 | yes | up | -2.01 | yes | down | hypothetical protein CICLE_v10033350mg [Citrus clementina] |
| **cds.TRINITY_DN105483_c0_g1_m.35990** | **CICLE90** | ORTHOMCL5482 | 0.722044245 | yes | up | -3.26 | no | down | hypothetical protein CICLE_v10026390mg [Citrus clementina] |
| **cds.TRINITY_DN119677_c3_g1_m.5655** | **HSPsmall** | ORTHOMCL18352 | 0.897481329 | yes | up | -1.78 | yes | down | PREDICTED: small heat shock protein, chloroplastic-like [Solanum tuberosum] |
| **cds.TRINITY_DN113183_c1_g3_m.25465** | **VITISV** | ORTHOMCL11613 | 0.757405126 | yes | up | -2.25 | yes | down | hypothetical protein VITISV_012137 [Vitis vinifera] |
| **cds.TRINITY_DN104690_c0_g1_m.30385** | **HSPV** | ORTHOMCL4992 | 0.416305007 | no | up | -6.47 | yes | down | 15.4 kDa class V heat shock protein [Medicago truncatula] |
| **cds.TRINITY_DN118581_c1_g1_m.57823** | **EUGRSUZ** | ORTHOMCL17122 | -0.036771563 | no | no change | 1.85 | yes | up | hypothetical protein EUGRSUZ_D02073 [Eucalyptus grandis] |
| **cds.TRINITY_DN113183_c1_g1_m.25469** | **HSPclassII** | ORTHOMCL11612 | 1.874820695 | yes | up | 0.87 | no | up | PREDICTED: 17.1 kDa class II heat shock protein-like [Malus domestica] |
| **cds.TRINITY_DN111945_c1_g1_m.61182** | **3KCoA1** | ORTHOMCL10484 | -0.044525311 | no | no change | 0.73 | no | up | 3-ketoacyl-CoA reductase 1 isoform 1 [Theobroma cacao] |
| **cds.TRINITY_DN113846_c5_g1_m.13204** | **HSP70P15** | ORTHOMCL12257 | -0.461280156 | yes | down | -0.55 | no | down | PREDICTED: heat shock 70 kDa protein 15-like [Solanum lycopersicum] |
| **cds.TRINITY_DN117660_c1_g1_m.29640** | **HSP90P3** | ORTHOMCL16086 | 0.236920715 | yes | no change | -1.91 | no | down | PREDICTED: hsp70-Hsp90 organizing protein 3 [Phoenix dactylifera] |
| **cds.TRINITY_DN115810_c0_g1_m.38007** | **Fes1A** | ORTHOMCL14221 | 0.422470257 | yes | up | -2.39 | yes | down | Fes1A [Theobroma cacao] |
| **cds.TRINITY_DN113029_c1_g1_m.40650** | **PRUPE** | ORTHOMCL11456 | -0.516008223 | yes | down | -0.59 | no | down | hypothetical protein PRUPE_ppa012538mg [Prunus persica] |
| **cds.TRINITY_DN105843_c0_g2_m.28734** | **HSP70mito** | ORTHOMCL5722 | 0.172863282 | yes | no change | -0.22 | no | down | PREDICTED: heat shock 70 kDa protein, mitochondrial [Phoenix dactylifera] |
| **cds.TRINITY_DN120415_c3_g1_m.14952** | **HSP83** | ORTHOMCL19162 | -0.098227918 | no | no change | -0.64 | no | down | PREDICTED: heat shock protein 83-like [Vitis vinifera] |
| **cds.TRINITY_DN116330_c4_g3_m.35906** | **HSC70P2** | ORTHOMCL14701 | 0.010715542 | no | no change | -1.2 | no | down | PREDICTED: heat shock cognate 70 kDa protein 2-like [Solanum tuberosum] |

**Table S4.** Differently expressed genes (DEGs) enriched in peroxisome and carbon metabolic pathways in Polish Spirit (PS) compared to Stolwijk Gold (SG)

| **Protein** | **Transcript** | **Abbrevi.** | **Description** |
| --- | --- | --- | --- |
| cds.TRINITY_DN117844_c1_g1_m.47440 | ORTHOMCL16305 | POD4 | PREDICTED: peroxidase 4 [Vitis vinifera] |
| cds.TRINITY_DN115825_c1_g2_m.58122 | ORTHOMCL14236 | GLUST | glutathione-S-transferase tau 1 [Hevea brasiliensis] |
| cds.TRINITY_DN108395_c0_g1_m.42312 | ORTHOMCL7487 | POD65 | PREDICTED: peroxidase 65 [Vitis hypothetical protein VITISV_013676 [Vitis vinifera] |
| cds.TRINITY_DN119573_c2_g1_m.60072 | ORTHOMCL18241 | NADQO | NADPH:quinone oxidoreductase [Arabidopsis thaliana] |
| cds.TRINITY_DN119142_c0_g1_m.65719 | ORTHOMCL17749 | 2OGO | 2-oxoglutarate (2OG) and Fe(II)-dependent oxygenase superfamily protein [Theobroma cacao] |
| cds.TRINITY_DN96720_c0_g1_m.50160 | ORTHOMCL16482 | GSTU17 | PREDICTED: glutathione S-transferase U17 isoform 1 [Vitis vinifera] |
| cds.TRINITY_DN117586_c0_g2_m.50305 | ORTHOMCL16012 | GO1 | PREDICTED: glycolate oxidase1, chloroplastic-like [Fragaria vesca subsp. vesca] |
| cds.TRINITY_DN108848_c1_g2_m.66744 | ORTHOMCL7824 | RPE3 | PREDICTED: ribulose-phosphate 3-epimerase, cytoplasmic isoform [Malus domestica] |
| cds.TRINITY_DN111615_c0_g1_m.847 | ORTHOMCL10183 | R5PI | PREDICTED: probable ribose-5-phosphate isomerase 3, chloroplastic [Cucumis melo] |
| cds.TRINITY_DN108758_c3_g1_m.45874 | ORTHOMCL7756 | rbcS | ribulose-1,5-bisphosphate carboxylase/oxygenase small subunit [Panax ginseng] |
| cds.TRINITY_DN113752_c0_g1_m.52167 | ORTHOMCL12173 | TPI2 | RecName: Full=Triosephosphate isomerase, cytosolic; Short=TIM; Short=Triose-phosphate isomerase [Coptis japonica] |
| cds.TRINITY_DN110697_c0_g1_m.41852 | ORTHOMCL9341 | FBP2 | PREDICTED: fructose-1,6-bisphosphatase, chloroplastic-like [Vitis vinifera] |
| cds.TRINITY_DN108328_c0_g1_m.9296 | ORTHOMCL7431 | ASAT1 | PREDICTED: aspartate aminotransferase, chloroplastic [Vitis vinifera] |
| cds.TRINITY_DN115048_c0_g1_m.59100 | ORTHOMCL13443 | PYK1 | PREDICTED: pyruvate kinase, cytosolic isozyme [Malus domestica] |
| cds.TRINITY_DN116230_c0_g2_m.46017 | ORTHOMCL14605 | 3PGD | d-3-phosphoglycerate dehydrogenase, putative [Ricinus communis] |
| cds.TRINITY_DN115840_c0_g2_m.838 | ORTHOMCL14250 | TS1 | threonine synthase [Lens culinaris] |
| cds.TRINITY_DN118838_c2_g3_m.31326 | ORTHOMCL17391 | DX5PR | 1-deoxy-D-xylulose 5-phosphate reductoisomerase [Actinidia arguta] |
| cds.TRINITY_DN112811_c0_g1_m.10404 | ORTHOMCL11260 | STK1 | serine-threonine kinase receptor-associated protein, putative [Ricinus communis] |
| cds.TRINITY_DN116752_c0_g1_m.42159 | ORTHOMCL15117 | STN8 | PREDICTED: serine/threonine-protein kinase STN8, chloroplastic [Vitis vinifera] |
| cds.TRINITY_DN174777_c0_g1_m.2568 | ORTHOMCL22134 | PPK2 | Os02g0698000 phosphoribulokinase, chloroplastic [Oryza sativa Japonica Group] |
| cds.TRINITY_DN81973_c0_g1_m.32532 | ORTHOMCL23232 | MDH2 | similarity to malate dehydrogenase precursor MDH - Mus musculus [Penicillium rubens Wisconsin 54-1255] |
| cds.TRINITY_DN109802_c2_g2_m.61429 | ORTHOMCL8559 | NADH2 | NADH dehydrogenase subunit 2 [Citrullus lanatus] |

**Table S5.** Expression levels of differently expressed genes in two *Cft* (PS and SG) in response to heat stress.

| Gene name | Clematis lines | Control | | Heat stress | |
| --- | --- | --- | --- | --- | --- |
|  |  | Mean | s.e. | Mean | s.e. |
| GLUST | Polish Spirit (PS) | 0.47871151 | 0.0334106 | 5.66068312 | 0.26243257 |
|  | Stolwijk Gold (SG) | 0.42432937 | 0.03193417 | 0.40414519 | 0.04794742 |
| GO1 | Polish Spirit (PS) | 0.57659822 | 0.03894752 | 4.15090352 | 0.48468661 |
|  | Stolwijk Gold (SG) | 0.58895664 | 0.04264118 | 1.73205081 | 0.26956684 |
| RPE3 | Polish Spirit (PS) | 0.39608124 | 0.02518109 | 2.77128129 | 0.27459335 |
|  | Stolwijk Gold (SG) | 0.35664153 | 0.02416538 | 1.15470054 | 0.23015317 |
| R5PI | Polish Spirit (PS) | 0.26243257 | 0.09148197 | 2.80237994 | 0.42432937 |
|  | Stolwijk Gold (SG) | 0.26956684 | 0.02866667 | 2.88675135 | 0.30845042 |
| rbcS | Polish Spirit (PS) | 0.48468661 | 0.02773941 | 3.81051178 | 0.38895664 |
|  | Stolwijk Gold (SG) | 0.43015317 | 0.03060636 | 1.99749844 | 0.33568049 |
| POD4 | Polish Spirit (PS) | 0.27459335 | 0.03917908 | 3.40445146 | 0.35664153 |
|  | Stolwijk Gold (SG) | 0.30845042 | 0.02278667 | 2.136196 | 0.27050637 |

**Supplemental Figures**


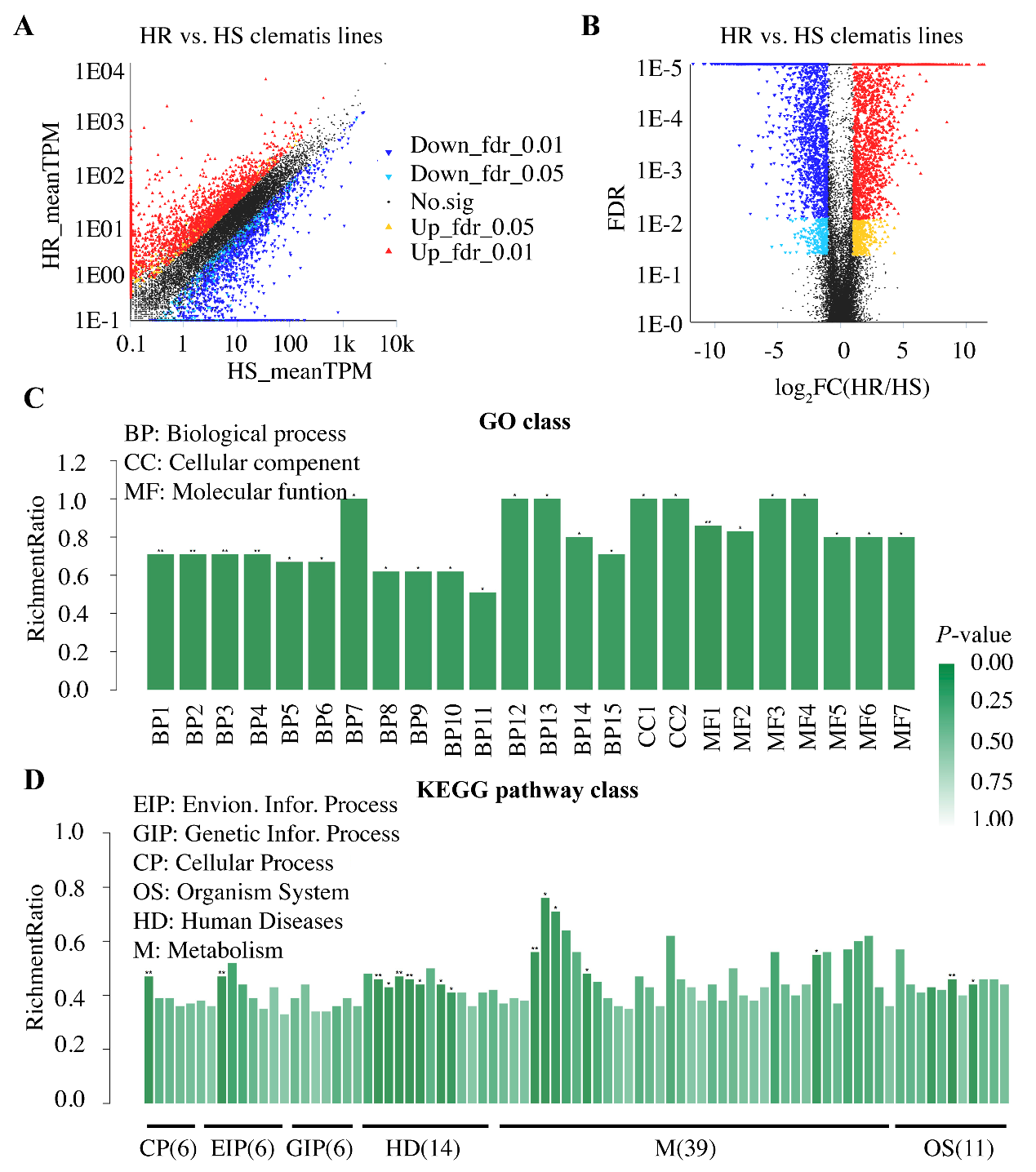


**Fig. S1.** Metabolic pathways and biological functions in global differencially expressed genes (DEGs) between two *Clematis florida* *thun* (*Cft)* lines (PS and SG) under heat stress versus normal condition. **A:** Correlation of DEGs between HR (PS) and HS (SG) lines. PS and SG represent heat-tolerant (HR) and heat-sensitive(HS)*Clematis florida* lines, respectively; **B:** Volcano plot representing False Detection Rates (FDR) of DEGs between HR and HS lines under heat stress against normal condition.. **C:** Gene ontology (GO) enrichments analysis on DEGs; **D:** Kyoto Encyclopedia of Genes and Genomes (KEGG) analysis on DEGs. P values were depicted in gradient green decreasing from 0 to 1. For GO pathway abbreviations (**C**). **BP1-BP15:** Negative regulation of RNA biosynthetic process, negative regulation of transcription, DNA-templated, negative regulation of NRA metabolic process, negative regulation of nucleic acid-templated transcription, negative regulation of cellular macromolecule biosynthetic process, aerobic respiration, regulation of nucleobase-containing compound metabolic process, negative regulation of biosynthetic process, flavonoid metabolic process, assembly of large subunit precursor of preribosome, phenylalany-tRNA aminoacylation, anthocyanin-containing compound biosynthetic process, chromatin silencing; **CC1-CC2:** protein serine/threonine phosphatase complex, phosphatase complex; **MF1-MF7:** solute:proton antiporter activity, solute:proton antiporter activity, monovalent cation: proton antiporter activity, phenylalanine-tRNA transporter activity, inorganic phosphate transmembrane transporter activity, xylosyltransferase activity, cation:cation antiporter activity. For KEGG pathway abbreviations (**D**): **CP1-CP6:** Apoptosis, Cell cycle-yeast, Meiosis-yeast, Cell cycle, ocyte meiosis, p53 signaling pathway; **EIP1-EIP6:** ABC transporters, NF-kappa B signaling pathway, TGF-beta signaling pathway, AMPK signaling pathway, Wnt signaling pathway, Plant hormone signal transduction; **GIP1-GIP6:** Sulfur relay system, Protein processing in endoplasmic reticulum, Homologous recombination, Non-homologous end-jointing, DNA replication, Spliceosome, RNA transport; **HD1-HD14:** Chemical carcinogenesis, Non-alcoholic fatty liver disease (NAFLD), Systemic lupus erythematosus, Pertussis, Tuberculosis, Leishmaniasis, Chagas disease (American trypanosomiasis), Toxoplasmosis, Amoebiasis, Meassies, Influenza A, Hepatitis B, Amyotrophic lateral scierosis (ALS), Alcoholism; **M1-M39:** Tyrosine metabolism, Cysteine and methionine metabolism, Tryptophan metabolism, Arginine biosynthesis, Phenylpropanoid biosynthesis, Flavonoid biosynthesis, Stibenoid, diarylheptanoid and gingerol biosynthesis, Tropane, piperidine and pyridine alkaloid biosynthesis, Starch and sucrose metabolism, Pentose and glucoronate interconversions, Glyoxylate and decarboxylase metabolism, Amino sugar and nucleotide sugar metabolism, Citrate cycle (TCA cycle), Nitrogen metabolism, Carbon fixation pathways in prokaryotes, Methane metabolism, Glycosphingolid biosynthesis -ganglio series, Glycosaminoglycan degradation, Lipopolysaccharide biosynthesis, Other glycan degradation, Alpha-Linolenic acid metabolism, Glycerophospholid metabolism, Ether lipid metabolism, Cutin, suberine and wax biosynthesis, Bio synthesis of unsaturated fatty acids, Steroid hormone biosynthesis, Folate biosynthesis, Biotin metabolism, Ubiquinone and other terpenoid-qunone biosynthesis, Riboflavin metabolism, Cynoamino acid metabolism, Taurine and hypotaurine metabolism, Glutathione metabolism, Monoteterpenoid biosynthesis, Ditepenoid biosynthesis, Brassinosteroid biosyntehsi, Drug metabolism-tytochrome P450, Metabolism of xenobiotics by Cytochrome P450; **OS1-OS11:** Regulation of lipolysis in adipocytes, Adipocytokine signaling pathway, Oxytocin signaling pathway, Plant-pathogen interaction, Circadian rhythm, Toll-like receptor signaling pathway, T cell receptor signaling pathway, Neurotrophin signaling pathway, GABAergic synapse, Retrograde endocannabinioid signaling, Glutamatergic synapse.

**
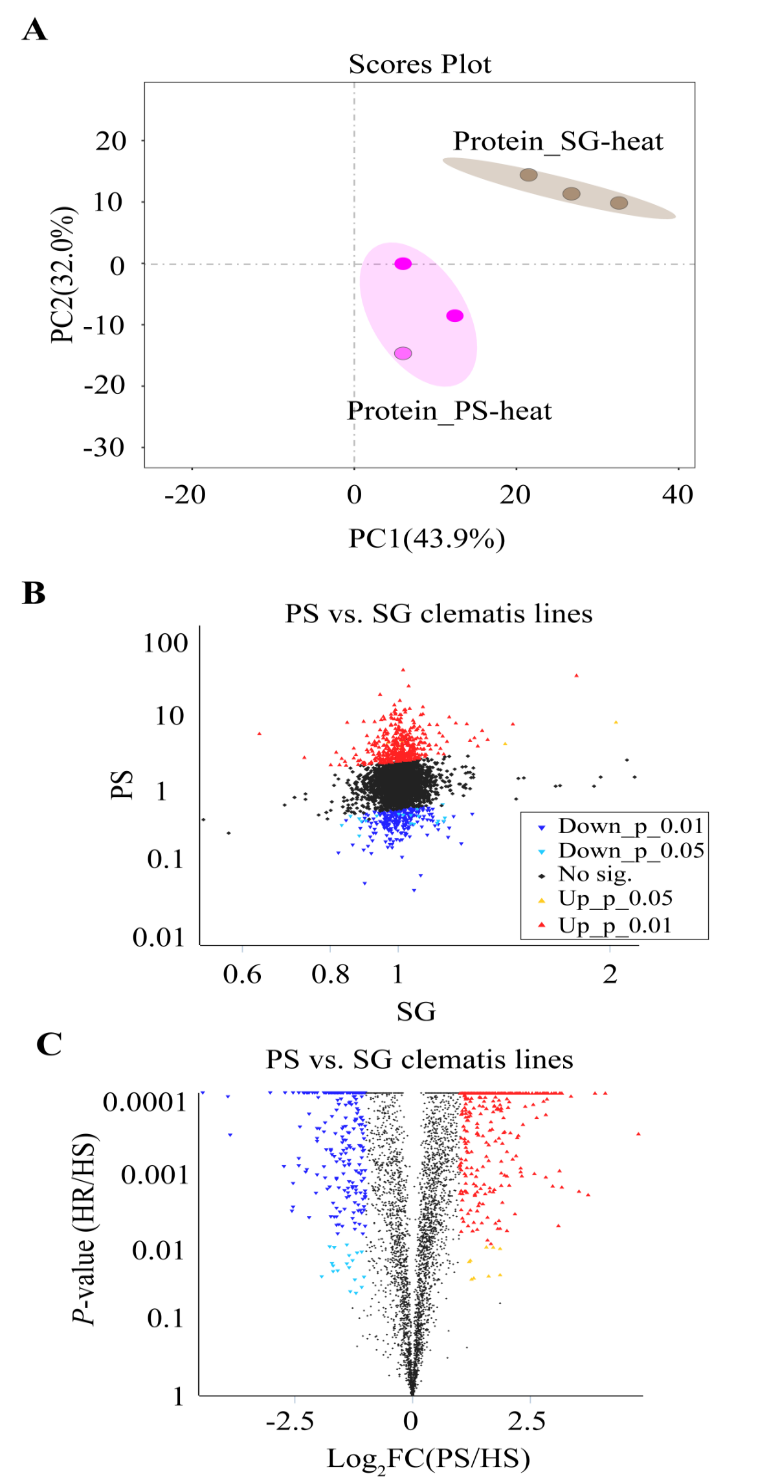
**

**Fig. S2.** Proteomics analysis on PS and SG under heat stress conditions. **A**: Principal components analysis on proteomics in PS and SG under heat stress conditions. **B-C**: Scatter and volcano plots of differentially expressed proteins (DEPs) in HR against HS lines of *Clematis florida thun* (*Cft*). HS and HR represent Clematis heat-sensitive and heat-tolerant lines, respectively.


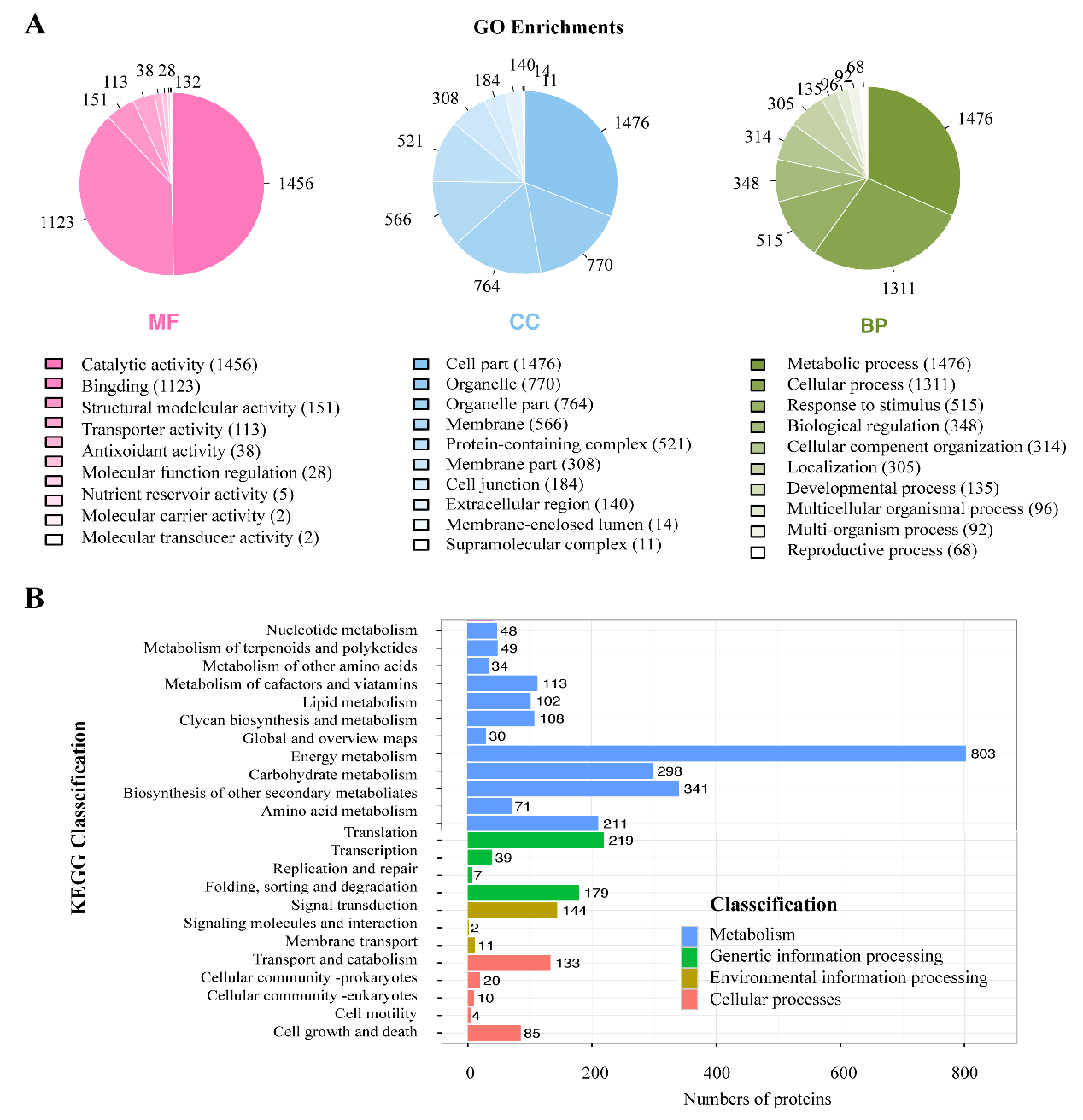
**Fig. S3.** GO and KEGG analyses of differentially expressed protein (DEPs) in *Clematis florida* *thun* (*Cft*) HR against HS. **A**: Gene ontology (GO) enrichments analysis on DEPs. **B**: Kyoto Encyclopedia of Genes and Genomes (KEGG) analysis on DEPs.
